# Bioinspired fractal electrodes for solar energy storages

**DOI:** 10.1038/srep45585

**Published:** 2017-03-31

**Authors:** Litty V. Thekkekara, Min Gu

**Affiliations:** 1 Laboratory of Artificial-Intelligence Nanophotonics, School of Science, RMIT University, Melbourne, Victoria 3001, Australia

## Abstract

Solar energy storage is an emerging technology which can promote the solar energy as the primary source of electricity. Recent development of laser scribed graphene electrodes exhibiting a high electrical conductivity have enabled a green technology platform for supercapacitor-based energy storage, resulting in cost-effective, environment-friendly features, and consequent readiness for on-chip integration. Due to the limitation of the ion-accessible active porous surface area, the energy densities of these supercapacitors are restricted below ~3 × 10^−3^ Whcm^−3^. In this paper, we demonstrate a new design of biomimetic laser scribed graphene electrodes for solar energy storage, which embraces the structure of Fern leaves characterized by the geometric family of space filling curves of fractals. This new conceptual design removes the limit of the conventional planar supercapacitors by significantly increasing the ratio of active surface area to volume of the new electrodes and reducing the electrolyte ionic path. The attained energy density is thus significantly increased to ~10^−1^ Whcm^−3^- more than 30 times higher than that achievable by the planar electrodes with ~95% coulombic efficiency of the solar energy storage. The energy storages with these novel electrodes open the prospects of efficient self-powered and solar-powered wearable, flexible and portable applications.

The emergence of solar energy storages^1^ open a cost-effective platform to overcome the issue of obtaining solar electricity irrespective of seasonal changes and enhances the possibilities to consider solar electricity as the major energy source in the future. Current research in this area has been mainly focused on generating solar energy storages physically separated from solar cells^2^,^3^. Developing integrable energy storages with flexible thin-film solar cells^4^ is desirable for environmental friendly solutions. The development of on-chip solar energy storage platforms^5^ integrated with laser scribed graphene micro-supercapacitors (LSG-MSCs) with interdigited electrodes are particularly promising for a broad range of applications in micro^6^ and bio-wearable electronics^7^, self-powered nano-piezo-electronics^8^,^9^ as well as future solar-powered applications if the energy density of MSCs can reach the level equivalent to lithium ion batteries^10^. The advancement of LSG-MSC fabrication from the sandwich^11^ to interdigited porous electrodes^12^,^13^,^14^, in conjunction with the development of solid-state ionic electrolytes, can increase the energy density up to ~3 × 10^−3^ Whcm^−3^ without the loss of a faster rate of charge-transfer performance^15^ However, the device performance is still not comparable with the conventional batteries^16^ because of the limit of the active area of planar porous electrodes and the long mean ionic free path of electrolytes^13^.

In this paper, we propose a new design concept of LSG-MSCs using bioinspired electrodes based on the ingenious fractal structures^17^ with broadened aspects for on-chip energy storage integrated thin-film amorphous silicon solar cells. Earlier related works using fractal families can be classified into optoelectronic transparent conductive electrodes^18^ and the study of mechanical properties of fractal designs in stretchable electronics^19^. The new design is based on the internal structure of natural fern leaves, *Polystichum munitum* (Fig. 1(a)), generally known as *Bransley fractals*^20^, which resemble the space filling curves of fractals with self-similar structures (Fig. 1(b–d))^21^,^22^. It is well known that Fern leaves are an efficient platform for energy storage in biological processes such as photosynthesis enabled by water transport on its vein density^23^,^24^ as well as information compression^25^.

## Results and Discussion

The space filling curves of fractals are a mathematical concept, based on the Cantor set designed by John Cantor in 1874, which define the curves that pass through every point of a n-dimensional region^26^. We can construct the space filling curve^26^ by considering a continuous function b from the Canter space *c* onto the entire unit interval {0, 1}. This results in the formation of continuous function B from the topological product *c x c* onto the whole unit square {0, 1} × {0, 1} by setting,


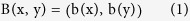


where the Canter space is written as 2^N^, where *2* denotes the 2-element set {0, 1} and can be defined as the infinite topological product of the discrete 2-point space {0, 1}.

**Figure 1:**
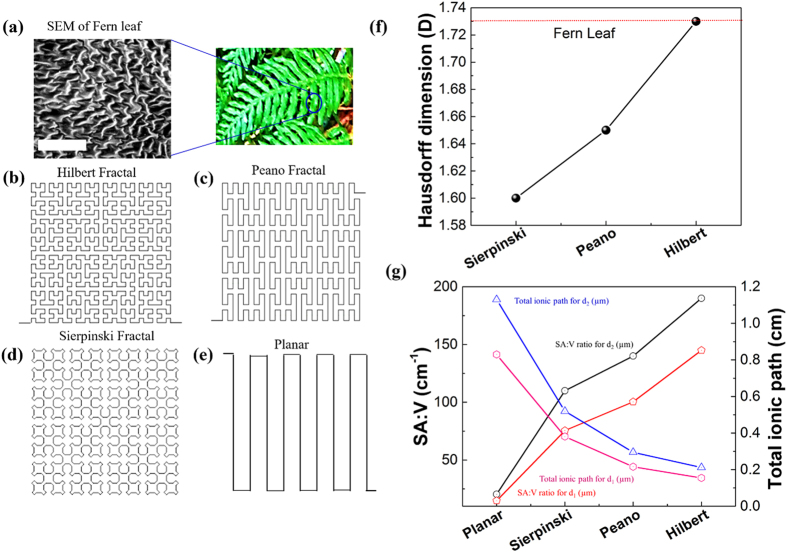
Bio-inspired fractal electrode designs. (**a**) Fern leaves (*Polystichum munitum*). Highlighted: SEM of the internal structure of Fern leaves. Scale bar 100 μm. (**b**) Hilbert fractal structures. (**c**) Peano fractal structures. (**d**) Sierpinski fractal structures. (**e**) Planar structures. (**f**) Calculated Hausdorff dimensions. (**g**) The surface area to volume ratio (SA:V) and the total ionic path obtained for different MSC electrode designs for two widths d_1_ and d_2_ in an area of 4 × 4 cm^2^.

In this study, we consider three cases in the space filling family, Hilbert fractals^27^ (Fig. 1(b)), Peano fractals^28^ (Fig. 1(c)) and Sierpinski fractals^29^ (Fig. 1(d)), with a non-fractal pattern (Fig. 1(e)) for comparison. The available storage area can be mathematically optimized using the dimension of the space filling curves and can be characterized by the Hausdorff dimension calculation^30^. The optimization of different fractal designs for MSCs requires the improvement of the available active area for the electrodes. These designs follow the linear equations in the iteration with a dimensionality represented by the Hausdorff dimension, D which is the measure of the local size of a set of numbers and can be calculated by Box-counting method^31^,^32^. The relationship between the Hausdorff dimension D^29^, the linear scaling L and the resulting increase in size, S for a two-dimensional (2D) object plane having length, width, and height be generalized and written as the equation given by:
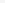



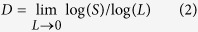


From the calculations, we find that the Hilbert fractals attain the highest dimension of 1.73 which is close to that found in the Fern leaves (Fig. 1(f)). These space filling fractal patterns form the design principle for bio-inspired fractal electrode micro-supercapacitors (BFE-MSCs).

**Figure 2:**
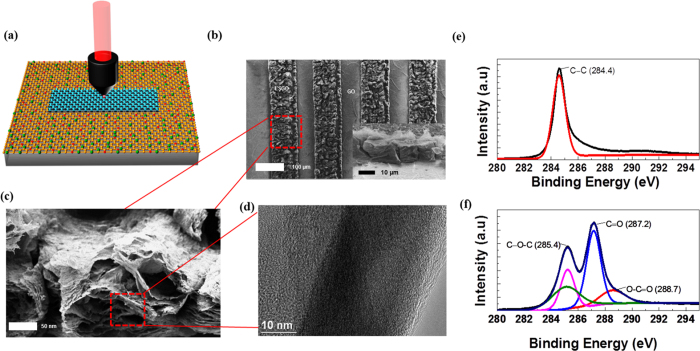
LSG films. (**a**) The CO_2_ laser beam of wavelength 10.6 μm for the LSG fabrication. Highlighted: porous LSG obtained at a laser power of 1.9 W. (**b**) SEM image of LSG patterns. Inset: the cross-sectional image of a LSG film of thickness 20 μm. (**c**) A high-resolution SEM image which shows the porous nature of the LSG film. (**d**) HR-TEM image of a LSG film, which indicates the crystalline nature with the mesoporous structures. (**e**) The XPS spectrum of the LSG film, which shows the removal of oxygen groups completely and formation of sp^2^ C-C bonds. (**f**) XPS spectrum of GO film.

The LSG method (Fig. 2(a)) is used to generate electrodes along the space filling curves, as demonstrated in Fig. S1. Apparently, the electrode density of a given fractal pattern is limited by the width of the filling curves determined by the size of the focal volume of the laser beam for LSG. In brief, the LSG films of a thickness, *t*, 20 μm (Fig. 2(b)) were obtained with a CO_2_ laser beam of wavelength 1064 nm^12^ with a lateral resolution of 80 μm for 0.25 numerical aperture (NA) objective and the details on the optimization of graphene oxide (GO) photoreduction parameters such as fine tuning of porous microstructure using the laser fluence can be found in our earlier paper^5^.

The thermogravimetric (TGA) studies under argon atmosphere ensures that the decomposition temperature of the obtained LSG film is above 1000 °C whereas that of GO is around 190 °C (Fig. S2(a)). The scanning electron microscopy (SEM) image of the resulting LSG film using a laser power of 1.9 W reveals a porous nature that in turn facilitates the penetration of electrolyte ions into the accessible surface areas (Fig. 2(c)). The pore size of the LSG film is studied using the Barnett-Joyner-Halenda (BJH) analysis^33^ and observed that the LSG porosity varies from 2 to 48 nm (Fig. S2(b)). It is well known that the pore size contributes to the current generation which influences the areal and volume capacitances in addition to the active surface area^15^ and that the smaller pore sizes contribute to the higher energy densities while, the larger pore sizes contribute to the higher power densities^34^,^35^. The high crystalline quality of the films is studied using the high-resolution transmission electron microscopy (HR-TEM) (Fig. 2(d)). The breakage and removal of oxygen bonds during the photoreduction of GO (Fig. 2(e)) is further confirmed by the XPS characterizations in the LSG film (Fig. 2(f)). The studies show the presence of dominant C-C peaks with the removal of C = O, C-O-C and O-C = O bonds of GO.

The dependence of the electrical conductivity on the volumetric electrode width d_1_ and d_2_ is determined from the four-probe measurements (Fig. S2(c)) which were 10^2^ S/m and 10^4^ S/m, respectively. We have therefore assigned the width, d_2_ of 300 μm, close to the maximum of the conductivity, in the MSC electrode fabrication. Numerical simulation studies show that the maximum temperature attained for GO layers during the laser irradiation process is around 1200 °C (Fig. S2(d)) which is sufficient to remove the oxygen groups present in the GO films^36^.

The performance of the BFE-MSC can be significantly enhanced by two physical mechanisms. The first mechanism is based on the enhancement of the effective electrode volume even though the space filling curves are planar structures. To illustrate this finding, let us consider two typical electrode widths (Fig. S2(c)), d_1_ and d_2_. The calculated geometric active surface area, *active area*_*geo*_, for a total area of 4 × 4 cm^2^ shows that the ratio of the active surface area between the two widths is the highest for Hilbert fractals, revealing a 36% improvement (Fig. S3). We thus calculated the ratio of active surface area to volume (SA: V) of the different electrode designs (Fig. 1(g)) by considering the pore size and thickness, *t* with widths d_1_ and d_2_ of the LSG films. In our calculations, we considered a mean pore size of 13.73 nm obtained from the BJH measurements^33^ in Fig. S2(b) and assumed that the pores are circular in shape. The second parameter considered is the active geometric area, *Active area*_*geo*_ obtained for different LSG-MSC electrodes with thickness, *t,* 20 μm as given in Fig. S3. The area of one pore, *A*_*pore*_ is given as


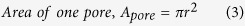


Using this equation, we can calculate the


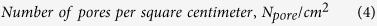


Based on this calculation we can further calculate the effective electrode active area; *Active area*_*eff*_ as given by:


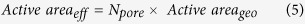


The effective active electrode volume, *Active volume*_*eff*_ can be calculated by taking into account the thickness, *t* of the LSG and the effective active area, *Active area*_*eff*_.


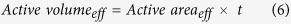


The ratio of the surface area to volume, *SA:V* in cm^−1^ is calculated (Fig. 1(g)) as follows:


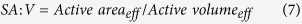


**Figure 3:**
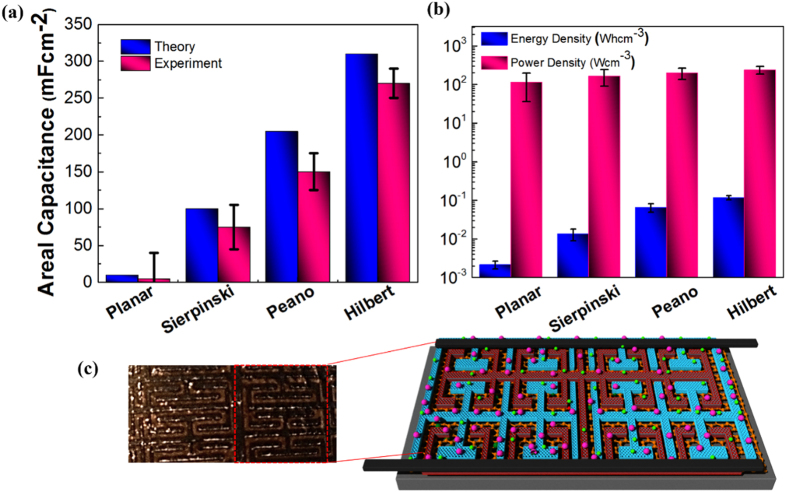
Comparison of different MSC designs. (**a**) Comparison of the areal capacitance between the theoretical and experimental calculation results for various BFE and planar MSCs. (**b**) Energy and power density calculations based on the volumetric capacitance obtained at a scan rate of 10,000 mVs^−1^ for each MSC of thickness 20 μm. (**c**) Image of a fabricated Hilbert BFE-MSC. Highlighted: Schematic of the Hilbert BFE-MSC structure.

From Fig. 1(g), we have shown that as electrode SA:V ratio improves it ultimately lead to higher energy densities and power densities (Fig. 3(d)). The results show an increase of SA:V by 10 times between the Hilbert BFE-MSC (Fig. S4) and the non-fractal MSC.

The second physical mechanism for enhancing the performance of the BFE-MSC is the electrolyte ion diffusion distance reduction compared to its planar counterpart. The dimensionality of the fractal design plays the key role in deciding the interdistance between two adjacent electrodes. In the case of the MSC fabrications, the width of electrodes and the size of electrolyte ions determine the maximum dimensionality that can be attained for the given fractal designs (Fig. S1). We calculated the electrolyte total ion diffusion length^37^ for electrode width, d_1_ and d_2_ (Fig. 1(g)) as follows:


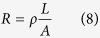


where *ρ* is the electrolyte resistivity^38^, *L* is the distance ion travels, *R* is the resistance offered by the device and *A* = total area-total active area of the device. The results confirm a decrease of 34% in the ion diffusion distance between the non-fractal MSC and the Hilbert BFE-MSC.

The theoretical aspect of the study is extended to understand the performance of different BFE-MSCs (Fig. S1) for a given area of 4 × 4 cm^2^. We chose the Stern-Gouy-Chapman model^39^ for the studies due to its simplicity in defining the double layer capacitance, C^40^ using the equation,


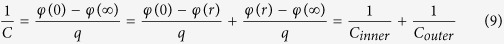


where 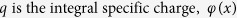 is the electric potential at a distance *x* in the medium to the interface and the *C*_*inner*_and *C*_*outer*_ are the capacitances formed in the medium and at the interface. The model predicts that the performance of supercapacitors can be influenced by the inner Stern layer which consists of immobile solvent ions and the diffuse outer layer comprised of mobile point charges. The theoretical analysis using an analytical equivalent circuit model^41^ shows that the enhancement of the areal capacitance is up to 300 times for Hilbert BFE electrodes compared to its planar electrode counterpart (Fig. 3(a)).

The electrolyte involved in the MSC fabrication is the ionic liquid 1-butyl-3-methyimidazolium bis (trifluoromethyl sulfonyl) imide which have an electrochemical window of 2.5 V^42^. The characterizations for different MSCs are conducted using electrochemical measurements, and further analysis is performed using the calculations given in references 12, 13, 14. The comparative study between the energy densities and power densities of different BFE-MSCs is shown in Fig. 3(b). The charging performance for different BFE-MSCs are verified from numerical simulations based on the equivalent circuit analysis described in the *Methods* and experiments using an applied voltage of 5 V with respect to time is studied (Fig. S5). A similar condition is used for the experimental studies using a standard DC charger of 5 V. An expected decrease in charging performance is observed due to the internal resistance offered by the current collectors. A co-relation upto 0.97 is calculated from Pearson co-efficient for Hilbert BFE-MSCs between theoretical and experimental studies.

The self-discharge phenomenon informs about the charge holding capacities of different MSCs. We studied the phenomenon in room conditions and compared with obtained output voltage and self-discharge resistive hours with equivalent series resistance (ESR) of the devices (Fig. S6). The new BFE-MSCs retained more ions due to the higher porous surface active area which leads to longer self-discharge resistive hours. ESR obtained from a charge-discharge, and impedance spectroscopy measurements provide information regarding the ion transport within the pores of electrode material. As observed, when the ESR reduces, the charging becomes faster with higher obtained output voltage and longer self-duration for the Hilbert BFE-MSCs.

**Figure 4:**
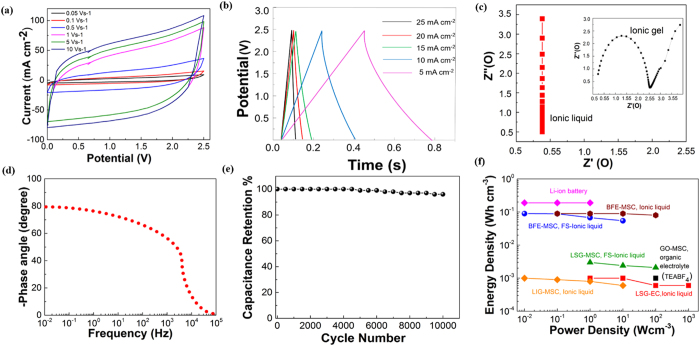
The performance of Hilbert BFE-MSC using ionic liquid electrolytes. (**a**) CV curves of the Hilbert BFE-MSC at different scan rates. (**b**) Galvanostatic charge-discharge (CC) curves of the Hilbert BFE-MSC at different charge densities. (**c**) Nyquist plot of the Hilbert BFE-MSC for different frequency scans using ionic liquid. Inset: Nyquist plot of Hilbert BFE-MSC using the ionic gel. The ESR of the optimized Hilbert BFE-MSC was confirmed to be 0.53 Ω. (**d**) Bode phase of the Hilbert BFE-MSC which gives the time relaxation constant of 0.273 ms using ionic liquid. (**e**) Capacitance retention of the Hilbert BFE-MSC up to 10,000 cycles for a scan rate of 5000 mVs^−1^. (**f**) Energy densities at corresponding power densities of the optimized Hilbert BFE-MSC are compared with other energy storages obtained from refs 11, 12, 13, 14.

Details of the electrochemical characterization of the optimized Hilbert BFE-MSC (Fig. 2(c)) are given in Fig. 4. The cyclic voltammetry (CV) measurements from scan rates of 50 mVs^−1^ to 10,000 mVs^−1^ (Fig. 4(a)) shows that the MSC maintained a nearly rectangular shape throughout the different scan rates, which ensures the faster charge transfer performance of the device. The galvanostatic charge-discharge studies from 5 to 25 mAcm^−2^ shows a triangular shape even at higher current densities with a small voltage drop (IR drop) of 0.53 V, calculated from the slope of charge-discharge curves (Fig. 4(b)). In addition to the increase of the active surface area, the reduction in the mean ionic free path of electrolyte ions in the case of fractal electrodes compared to the planar electrodes is confirmed using, electrochemical impedance spectroscopy (EIS) measurements conducted a frequency range of 10 Hz to 100 kHz. The comparison between the performances is studied using both ionic liquid and ionic gel electrolytes as given in Fig. 4(c) and observed that the ionic gel degrades the performance compared to the ionic liquid which seems to be the contributed from the presence of silica particles in the gel. The capacitative behavior of different designs is obtained from the intercept on the real axis of complex-plane impedance plot (Fig. 4(d) and the effective series resistance (ESR) of the optimized Hilbert BFE-MSC is around 0.15 Ω due to the highly available surface of LSG for the electrolyte ions. The calculated RC time constant of Hilbert BFE-MSCs is around 8.6 ms.

The superior performance of the obtained BFE-MSC is tested from the cyclic stability of supercapacitors with different electrode designs and observed that the Hilbert supercapacitors retained 95% of capacitance even after 10,000 charge/discharge cycles (Fig. 4(e)). The enhancement of ion accessible electrochemical surface area can explain the improved performances, resulting in the minimization of the ion diffusion pathway from the electrolyte to the electrode material. It should be noted that the energy density of the Hilbert BFE-MSC is 30 times better than the reported LSG-MSCs^11^,^12^,^13^,^14^ and close to Li-ion batteries (Fig. 4(f)). Advanced super-resolution nanofabrication technique might bridge this gap^43^.

**Figure 5:**
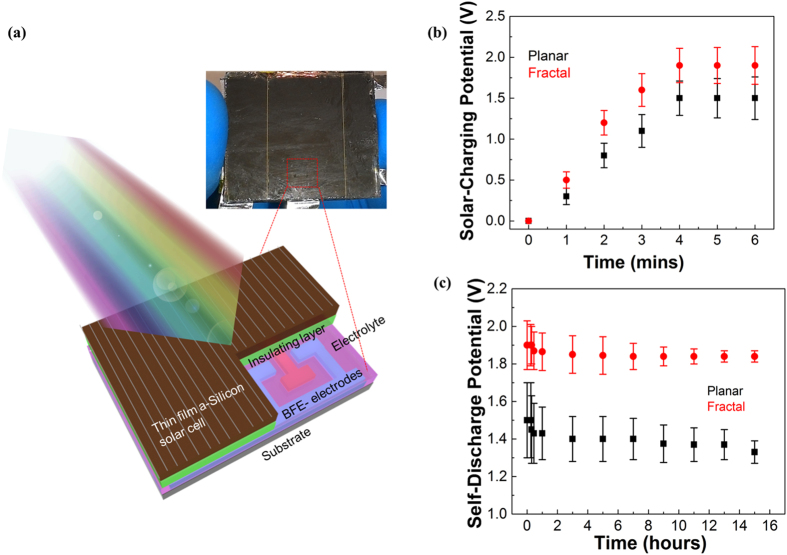
The performance of the Hilbert BFE-MSC integrated thin film amorphous silicon solar cell. (**a**) Schematic of Hilbert BFE-MSC integrated thin film amorphous silicon solar cell under one sun illumination. (**b**) Solar charging performance of integrated planar and BFE MSCs. (**c**) The self-discharge performance of the integrated BFE-MSC and planar MSC.

We further demonstrated an on-chip integration of the high-performance Hilbert BFE-MSCs and thin film amorphous silicon (a-Si) solar cells of efficiency 10% as shown in Fig. 5(a) (*See Methods*). We used an insulator of SU-8 in-between the solar cells and the GO layer, which avoids the influence of laser fluence on the solar cell and performed control experiments using thin film a-Si solar cell before and after the BFE-MSC integration (Fig. S7(a)). Galvanostatic charge-discharge studies are conducted to understand the performance of integrated energy storage using a current of 24 mA and non-degraded performance until 800 cycles are observed as shown in Fig. S7(b). The solar charging attained under One Sun (1000 W/m^2^) condition in the room temperature is around 2 V for integrated Hilbert BFE-MSCs (Fig. 5(b)) (see Methods). The columbic efficiency which is the ratio between charge in the MSC during the discharge cycle to the existing charge during the charge cycle, is around 95% with self-discharge hold potential of more than 14 hours (Fig. 5(c)).

All solid-state Hilbert, BFE-MCs is fabricated on a flexible platform such as polyethylene terephthalate (PET) using ionic gel electrolyte as shown in Fig. S8. The possibilities of performance loss are tested by bending up to 180 °C (Fig. S8(b)) and twisting upto 90 °C (Fig. S8(c)). The cyclic voltammetry is conducted under different bending, and twisting conditions (Fig. S8(d)) at a scan rate of 5000 Vs^−1^ and observed that the nearly rectangular shape is maintained without degradation. The superior performance of the flexible supercapacitor is tested from the cyclic stability of supercapacitors and observed capacitance retention of 90%, even after 10,000 charge/discharge cycles (Fig. S8(e)). These results open a pathway for meeting the demands of the current technology like self-powered graphene energy storages for wearables and various self solar energy-powered devices, which will have a significant impact in various areas of human society.

## Methods

### Materials

Modified Hummer’s method^44^ was used to synthesize the GOs. We used two types of electrolytes: (1-butyl-3-methylimidazolium bis (trifluoromethyl sulfonyl) imide {[BMIM] [NTf_2_]} (Sigma-Aldrich) ionic liquid and ionic gel by adding fumed silica of 7 nm in size (Sigma-Aldrich) to the respective ionic liquid.

### Material Synthesize

1.3 mg/ml of GOs was dispersed in water and drop cast on a glass substrate. It could dry under ambient conditions for 24 hours. The ionogel was prepared by mixing fumed silica nanopowder with the ionic liquid (1-butyl-3-methylimidazolium bis (trifluoromethyl sulfonyl) imide {[BMIM] [NTf_2_]}in a ratio 0.03 g/1.0 g and the mixture was stirred under a nitrogen atmosphere for about 5 hours. The electrochemical window of the electrolyte is 2.5 V.

### Fabrication of bioinspired fractal electrode micro-supercapacitors (BFE-MSC)

With the computer-assisted laser scribing using CO_2_ laser beam (Versa laser) of wavelength 10.6 μm, we fabricated the laser scribed graphene (LSG) electrodes. We found that BFE–MSC using drop-cast films gave the best performance in comparison with films prepared by other methods. The threshold power for laser scribing of the drop-cast GO film was 1.9 W, as obtained from the reference^5^.

### Theoretical Model

The theoretical considerations included in this work were studied using the Gouy-Chapman-Stern standard model used for double layer capacitance at a metal/ionic liquid interface^45^,^46^,^47^. The model considers an inner atomic dimension “Helmholtz layer” which is a charge free and outer charged “diffuse layer or Gouy-Chapman layer”.

### Numerical Model

Simulation based on the equivalent circuit model^41^ was performed using Matlab. In the simulation, we considered the following conditions:

- Internal resistance to be constant during the charge and discharge cycles.
- The temperature effect of the electrolyte and the aging effect of the device was not considered.
- Current through the supercapacitor was continuous.
- Charge redistribution is same for all values of voltage.

### Electrochemical measurements

The electrochemical measurements which include cyclic Voltammetry with a step size of 0.1 V from 0 to 2.5 V, galvanostatic charge-discharge and impedance spectroscopy in a frequency range of 10 KHz-10 Hz were conducted using potentiostat station (Autolab 100) at room temperature. The device volume includes two LSG current collector electrodes, LSG planar or fractal electrodes and the separator. We followed references^13^,^14^ for the calculations of energy and power densities based on these measurements.

In brief, the specific capacitance was calculated from galvanostatic (CC) curves at different current densities by the formula:


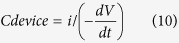


where *i* is the applied current (in amps, A) and *dV/dt* is the slope of the discharge curve (in volts per second, V/s).

Volumetric capacitance was given by


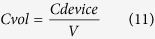


where A and V refer to the area (cm^2^) and volume (cm^3^).

The power density of the device was calculated from galvanostatic curves at different charge/discharge densities and given by the formula:


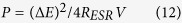


where P is the power in *W/cm*^3^, Δ*E* is the operating voltage window and *R*_*ESR*_ if the internal resistance of the device and can be given by the formula:

The energy density of the device can be calculated by the formula:


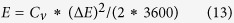


where E is the energy density in *Wh/cm*^3^, *C*_*v*_ is the volumetric capacitance and is the operating voltage window, V.

### Numerical simulations for GO surface heating during laser irradiation

We conducted a theoretical study for detailed understanding of the temperature profile of GO film during continuous wave (CW) laser irradiation process of wavelength 1064 nm using Comsol Multiphysics software based on finite element method (FEM) by solving the heat balance equation^48^. The thickness of each layer of film was 1.5 nm on a silica glass substrate. The boundary condition was selected as thermally insulating in all directions to consider thermal conductivities of air and GO. The boundary condition was selected as thermally insulating in all directions to consider thermal conductivities of air and GO. If the laser radiation is spatially uniform, the absorbed laser energy is instantaneously converted into the local heat, which can diffuse by thermal conduction and heat conduction and written as *T (X, t*) at the depth *X* and time *t* as


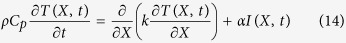


where ρ is the mass density, C_p_ is the specific heat, 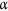 is the absorption coefficient and k is the thermal conductivity. The laser power density I *(X, t)* is determined by the interaction of the laser radiation with GO film and the subsequent transfer of the energy to the lattice. The laser power density can be


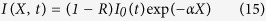


where R is the reflectivity and I_0_ (t) is the temporal distribution of the laser power.

In simulations, we considered the same laser power in the experiments which was conducted in room conditions with initial temperature across the surfaces as 25 °C. We considered up to 10 layers in the simulation studies to obtain the temperature profile with extremely fine meshing conditions. The material parameters used for the simulation are of amorphous carbon^49^ to obtain a reasonable temperature. The thermal conductivity of GO and silica glass was considered as isotropic. An assumption was made by considering absorption co-efficient of GO at 1064 nm as constant irrespective of several layers.

### Fabrication of energy storage integrated thin-film silicon solar cells

We followed the method described in our earlier paper^5^ for the fabrication of on-chip energy storage integrated solar cells. The aluminum tapes were used as current collectors as shown in Fig. 4(a). In short, current collectors were attached to the reverse side of the P-I-N thin film a-Si solar cell, and the aluminum tape was used has current collectors from BFE-MSC electrodes with in between adhesive as the silver paste to improve the conductivity. GOs was deposited on the reverse side of solar cell which had an insulating layer of SU8 resist, and the laser scribing of BSE pattern was performed at a power of 1.9 W. Finally, glass substrates were used to encapsulate the integrated device to make it portable.

The solar cell performance was analyzed before and after the encapsulation. In addition, the studies were extended during different time intervals of the solar charging process (Fig. S7(a)). The solar charging measurements were studied by a solar simulator (Oriel 3A) under a One-Sun condition (1000 W/m^2^) at the room temperature. The aluminum tapes were connected between solar cell and energy storage, so that charge generated in solar cell will be simultaneously charged the energy storage, and a saturation stage was observed depending on the capacity of energy storage to store the charge as well as the ability of the solar cell to generate the charge under the longer light exposure times, and the self-discharge was studied in the atmospheric conditions. The columbic efficiency was calculated by the formula,


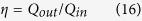


where *Q*_*out*_ is the amount of charge that exists in the supercapacitor during the discharge cycle and *Q*_*in*_ is the amount of charge that exists in the supercapacitor during the charge cycle. The galvanostatic charge-discharge studies were conducted at an applied current of 0.03A which shows an excellent stability of an integrated energy storage device (Fig. S7(b)).

## Additional Information

**How to cite this article:** Thekkekara, L. V. and Gu, M. Bioinspired fractal electrodes for solar energy storages. *Sci. Rep.* **7**, 45585; doi: 10.1038/srep45585 (2017).

**Publisher's note:** Springer Nature remains neutral with regard to jurisdictional claims in published maps and institutional affiliations.

## Supplementary Material

Supplementary Information
