# Supplementary material for: Bioinspired fractal electrodes for solar energy storages
Source: Sci Rep. 2017 Mar 31;7:45585. doi: 10.1038/srep45585 (PMC5374533; doi:10.1038/srep45585)
Supplement: Supplementary Information [file srep45585-s1.docx]

Supplementary Materials for

**Bioinspired fractal electrodes for solar energy storages**

Litty V. Thekkekara, and Min Gu^1^*

*Correspondence to: min.gu@rmit.edu.au.

**This File includes**

Figs. S1 to S8


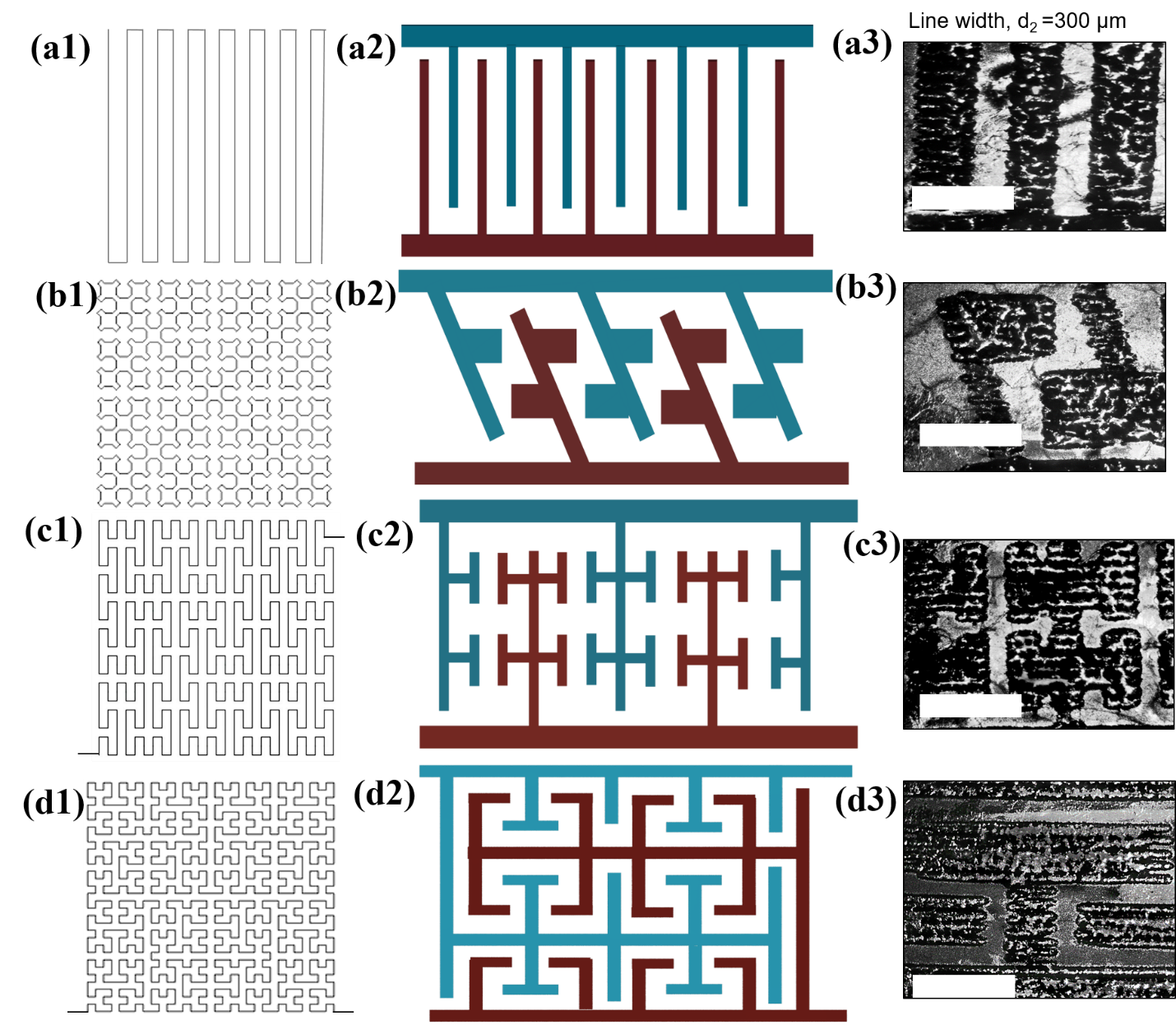


**Fig. S1. BFE-MSC designs using different space filling curves.** The first column shows the space filling curves, the second column shows the design of the interdigited electrodes and the third column shows the images of the fabricated electrodes (scale bar: 1 mm). (a1-a3) Planar design. (b1-b3) Sierpinski space filling design. (c1-c3) Peano space filling design. (d1-d3) Hilbert space filling design.


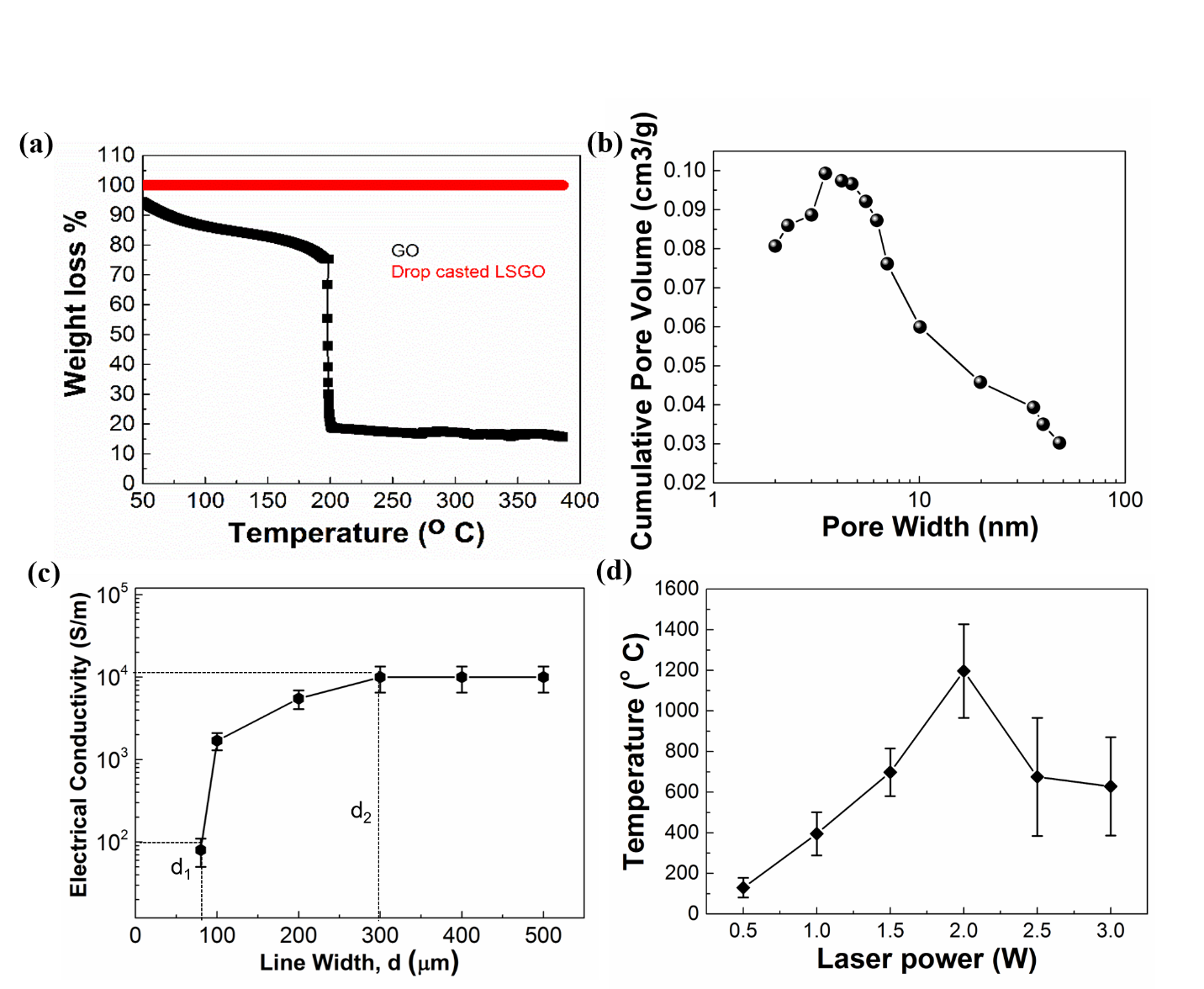


**Fig. S2. Characterizations of the LSG film.** (a) Thermogravimetric analysis (TGA) measurements done on the LSGO films which confirmed 100% reduction of LSGO films. (b) Porous analysis of LSG obtained using a laser power of 1.9 W from the BJH method. The pore size distributions vary from 2 to 48 nm. (c) The four-probe electrical conductivity measurement for the LSG films of different widths (two typical widths d_1_ and d_2_ are marked). The breakdown point was attained for GO films of the width below 200 µm, which resulted in the reduction of the electrical conductivity [50]. (d) The temperature profile of the LSG film using a CW laser irradiation of wavelength 1064 nm under various powers.





**Fig. S3. Geometric surface active area calculated for different BFE-MSC designs for electrode widths d_1_ and d_2_.**


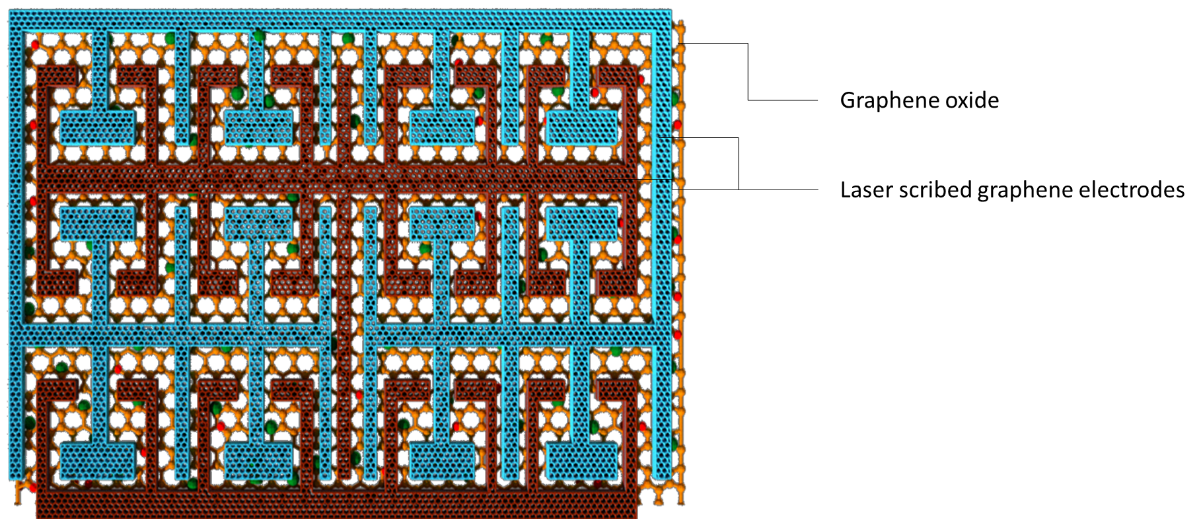


**Fig. S4. Schematic of porous Hilbert BFE supercapacitors.**

**Fig. S5. Charging performance of different BFE designs**. Numerical simulations were conducted using Matlab by using the standard model of the Stern-Gouy-Chapman and comparison was made between the charging performances obtained under experimental conditions using a standard DC charger of 5 V for (a) planar electrodes, (b) Sierpinski electrodes, (c) Peano electrodes and (d) Hilbert electrodes.

**

**

**Fig. S6. Comparison between the obtained equivalent series resistance, output operating voltage and self-discharge duration obtained for different electrode designs.** The equivalent series resistance was obtained from the impedance spectroscopic measurements on each BFE-MSC. The output voltage was calculated by charging the MSCs using a commercially available DC charger at a voltage of 5 V. The self-discharge studies were conducted under normal atmospheric conditions.

**Fig. S7. BFE-MSC integrated with a thin-film silicon solar cell.** (a) Thin-film a-Si solar cell performance before and after the integration of the Hilbert BFE-MSC. (b) Galvanostatic charge-discharge studies conducted on the Hilbert BFE-MSC integrated with the thin-film a-Si solar cell.

**Fig. S8. The performance of the flexible Hilbert BFE-MSC using ionic gel.** (a) Image of the flexible Hilbert BFE-MSC device. (b) Image of the BFE-MSC under a bent of 60 °C. (c) Image of the supercapacitor under the twisted condition of 60 °C. (d) CV curves of the Hilbert BFE-MSC at 5000 mVs^-1^ under flat, bent and twisted conditions. (e) Capacitance retention of the Hilbert BFE-MSC for 10,000 cycles under flat, bent and twisted conditions.

**References**
50. Qi, Z. J. et al. Electronic transport of recrystallized freestanding graphene nanoribbons. ACS Nano 9, 3510–3520 (2015).
